# Functional and Safety Outcomes of Carotid Artery Stenting and Mechanical Thrombectomy for Large Vessel Occlusion Ischemic Stroke With Tandem Lesions

**DOI:** 10.1001/jamanetworkopen.2023.0736

**Published:** 2023-03-01

**Authors:** Mudassir Farooqui, Osama O. Zaidat, Ameer E. Hassan, Darko Quispe-Orozco, Nils Petersen, Afshin A. Divani, Marc Ribo, Michael Abraham, Johanna Fifi, Waldo R. Guerrero, Amer M. Malik, James E. Siegler, Thanh N. Nguyen, Sunil Sheth, Albert J. Yoo, Guillermo Linares, Nazli Janjua, Milagros Galecio-Castillo, Wondewossen G. Tekle, Victor M. Ringheanu, Marion Oliver, Giana Dawod, Jessica Kobsa, Ayush Prasad, Asad Ikram, Eugene Lin, Kristine Below, Cynthia B. Zevallos, Marta Olivé Gadea, Abid Qureshi, Andres Dajles, Stavros Matsoukas, Ameena Rana, Mohamad Abdalkader, Sergio Salazar-Marioni, Jazba Soomro, Weston Gordon, Juan Vivanco-Suarez, Charoskhon Turabova, Maxim Mokin, Dileep R. Yavagal, Mouhammad A. Jumaa, Santiago Ortega-Gutierrez

**Affiliations:** 1 Department of Neurology, University of Iowa Hospitals and Clinics, Iowa City; 2 Department of Neurology, Saint Vincent Mercy Hospital, Toledo, Ohio; 3 Department of Neurology, Valley Baptist Medical Center/University of Texas Rio Grande Valley, Harlingen; 4 Department of Neurology, Yale University School of Medicine, New Haven, Connecticut; 5 Department of Neurology, University of New Mexico Health Science Center, Albuquerque; 6 Department of Neurology, Hospital Vall d’Hebron, Barcelona, Barcelona, Spain; 7 Department of Neurology, University of Kansas Medical Center, Kansas City; 8 Department of Neurosurgery, Icahn School of Medicine at Mount Sinai, New York, New York; 9 Department of Neurology and Brain Repair, University of South Florida, Tampa; 10 Department of Neurology, University of Miami Miller School of Medicine, Miami, Florida; 11 Cooper Neurological Institute, Cooper University Hospital, Camden, New Jersey; 12 Department of Neurology, Boston Medical Center, Boston, Massachusetts; 13 Department of Neurology, UTHealth McGovern Medical School, Houston, Texas; 14 Texas Stroke Institute, Dallas-Fort Worth; 15 Department of Neurology, Saint Louis University School of Medicine, St Louis, Missouri; 16 Asia Pacific Comprehensive Stroke Institute, Pomona Valley Hospital Medical Center, Pomona, California; 17 Department of Clinical Research, Valley Baptist Medical Center, Harlingen, Texas; 18 Department of Neurology, University of Toledo College of Medicine and Life Sciences, Toledo, Ohio; 19 Department of Neurology, Weill Cornell Medicine, New York, New York; 20 Department of Neurology, ProMedica Toledo Hospital, Toledo, Ohio

## Abstract

**Importance:**

Approximately 10% to 20% of large vessel occlusion (LVO) strokes involve tandem lesions (TLs), defined as concomitant intracranial LVO and stenosis or occlusion of the cervical internal carotid artery. Mechanical thrombectomy (MT) may benefit patients with TLs; however, optimal management and procedural strategy of the cervical lesion remain unclear.

**Objective:**

To evaluate the association of carotid artery stenting (CAS) vs no stenting and medical management with functional and safety outcomes among patients with TL-LVOs.

**Design, Setting, and Participants:**

This cross-sectional study included consecutive patients with acute anterior circulation TLs admitted across 17 stroke centers in the US and Spain between January 1, 2015, and December 31, 2020. Data analysis was performed from August 2021 to February 2022. Inclusion criteria were age of 18 years or older, endovascular therapy for intracranial occlusion, and presence of extracranial internal carotid artery stenosis (>50%) demonstrated on pre-MT computed tomography angiography, magnetic resonance angiography, or digital subtraction angiography.

**Exposures:**

Patients with TLs were divided into CAS vs nonstenting groups.

**Main Outcomes and Measures:**

Primary clinical and safety outcomes were 90-day functional independence measured by a modified Rankin Scale (mRS) score of 0 to 2 and symptomatic intracranial hemorrhage (sICH), respectively. Secondary outcomes were successful reperfusion (modified Thrombolysis in Cerebral Infarction score ≥2b), discharge mRS score, ordinal mRS score, and mortality at 90 days.

**Results:**

Of 685 patients, 623 (mean [SD] age, 67 [12.2] years; 406 [65.2%] male) were included in the analysis, of whom 363 (58.4%) were in the CAS group and 260 (41.6%) were in the nonstenting group. The CAS group had a lower proportion of patients with atrial fibrillation (38 [10.6%] vs 49 [19.2%], *P* = .002), a higher proportion of preprocedural degree of cervical stenosis on digital subtraction angiography (90%-99%: 107 [32.2%] vs 42 [20.5%], *P* < .001) and atherosclerotic disease (296 [82.0%] vs 194 [74.6%], *P* = .003), a lower median (IQR) National Institutes of Health Stroke Scale score (15 [10-19] vs 17 [13-21], *P* < .001), and similar rates of intravenous thrombolysis and stroke time metrics when compared with the nonstenting group. After adjustment for confounders, the odds of favorable functional outcome (adjusted odds ratio [aOR], 1.67; 95% CI, 1.20-2.40; *P* = .007), favorable shift in mRS scores (aOR, 1.46; 95% CI, 1.02-2.10; *P* = .04), and successful reperfusion (aOR, 1.70; 95% CI, 1.02-3.60; *P* = .002) were significantly higher for the CAS group compared with the nonstenting group. Both groups had similar odds of sICH (aOR, 0.90; 95% CI, 0.46-2.40; *P* = .87) and 90-day mortality (aOR, 0.78; 95% CI, 0.50-1.20; *P* = .27). No heterogeneity was noted for 90-day functional outcome and sICH in prespecified subgroups.

**Conclusions and Relevance:**

In this multicenter, international cross-sectional study, CAS of the cervical lesion during MT was associated with improvement in functional outcomes and reperfusion rates without an increased risk of sICH and mortality in patients with TLs.

## Key Points

**Question:**

Is carotid artery stenting (CAS) during mechanical thrombectomy (MT) associated with improved functional outcome without an increase in hemorrhagic rates in patients with large vessel occlusion ischemic stroke with tandem lesions, defined as concomitant large vessel occlusion and stenosis or occlusion of the cervical internal carotid artery?

**Findings:**

In this cross-sectional study of 685 patients with tandem lesions, compared with nonstenting, CAS of the cervical lesion during MT was associated with increased odds of achieving a modified Rankin Scale score of 0 to 2, whereas the rates of symptomatic intracranial hemorrhage were similar at 90 days.

**Meaning:**

These findings suggest that CAS of the cervical lesion during MT may represent an appropriate endovascular approach for patients with tandem lesions.

## Teaser

This cross-sectional study evaluates the clinical and technical outcomes of carotid artery stenting vs no stenting during mechanical thrombectomy in patients with acute large vessel occlusion strokes and concomitant tandem lesions.

## Introduction

Tandem lesions (TLs) involve intracranial large vessel occlusion (LVO) and concomitant stenosis or occlusion of the cervical internal carotid artery (ICA). Tandem lesions constitute approximately 10% to 20% of all LVO strokes.^1^ Treatment of TLs is challenging, with lower recanalization rates, poor prognosis, severe disability, and increased mortality.^2,3^ Mechanical thrombectomy (MT) was recently found to be beneficial in patients with LVO acute ischemic stroke with TLs.^4^ However, there is uncertainty in the optimal management of the cervical lesion, and the best technical strategy remains controversial.^4,5^

Endovascular revascularization treatment in patients with TLs varies based on clinical and technical complexities and proceduralist expertise. Some interventionists prefer to deploy a carotid stent for immediate recanalization during the MT procedure, either before (anterograde approach) or after (retrograde approach) intracranial thrombectomy. Others advocate for balloon angioplasty, aspiration of the cervical segment, or intracranial MT alone, which is then followed by a delayed treatment of the cervical lesion by endarterectomy, carotid artery stenting (CAS), or medical management in the subsequent days or weeks. Observational cohort studies^6,7,8,9^ published to date have reported conflicting results. Although some studies^6,7^ report benefits of CAS with favorable outcomes, others^8,9^ observed no differences in successful revascularization, clinical outcomes, or mortality after CAS.

Additional complexity in treatment decision-making occurs because CAS requires the use of antithrombotic medications, which can increase the risk of symptomatic intracranial hemorrhage (sICH).^10^ On the other hand, previous reports^7,11,12^ have observed the feasibility and beneficial results of CAS with no differences in hemorrhagic complications.

This inexplicit evidence exemplifies the necessity of further research to optimize the treatment approach for patients with TLs. We sought to evaluate the clinical and technical outcomes of CAS vs no stenting during MT in patients presenting with acute LVO stroke with TLs in a large multicenter collaboration.

## Methods

### Study Design, Settings, and Participants

This cross-sectional study used data from an international, retrospective, observational registry from 17 stroke centers (16 hospitals in the US and 1 in Spain). The population consists of consecutive patients with anterior circulation TLs treated with endovascular therapy within 24 hours after symptom onset, between January 1, 2015, and December 31, 2020. Inclusion criteria were age of 18 years or older, endovascular therapy for intracranial occlusion, and presence of extracranial ICA lesion on admission computed tomography angiography, magnetic resonance angiography, and/or intraprocedural digital subtraction angiography. Patients with isolated extracranial ICA lesions were excluded. The data collection instrument was previously prepared and shared with the site investigators to ensure the uniformity of the data collection. Additional variables were abstracted using the medical records. Tandem lesions were defined as an intracranial LVO (petrous, cavernous, or terminus segment of the ICA or M1 or proximal M2 segment of the middle cerebral artery) with a concomitant extracranial ICA stenosis of 50% or more and/or occlusion, as defined by the North American Symptomatic Carotid Endarterectomy Trial criteria.^13^ Because of the retrospective study design, this study was approved under a waiver of informed consent by the local institutional review boards at each participating center and is reported in accordance with the Strengthening the Reporting of Observational Studies in Epidemiology (STROBE) guidelines.^14^

### Study Groups, Data Elements, Exposures, and Interventions

Patients were divided into 2 groups according to the treatment strategy: (1) CAS group (patients treated with stenting of the cervical lesion during MT) and (2) nonstenting group (patients treated with balloon angioplasty or thrombectomy with thromboaspiration and/or stent retriever, only aspiration of the extracranial ICA lesion, or deferred or no extracranial ICA intervention). Information on patients’ demographic characteristics, risk factors and comorbid conditions, National Institutes of Health Stroke Scale (NIHSS) scores, modified Rankin Scale (mRS) scores, smoking status, history of antithrombotic treatment, and intravenous thrombolysis administration were abstracted from the medical record review. Imaging characteristics included baseline Alberta Stroke Program Early CT Score (ASPECTS), etiology of the extracranial ICA lesion, occlusion site (as determined on computed tomography angiography or digital subtraction angiography), and degree of stenosis and lesion type (as determined on digital subtraction angiography). Stroke workflow time metrics were time from last known well (LKW) to groin puncture, door to arterial puncture, and arterial puncture to reperfusion. Procedural variables included intracranial stenting and/or angioplasty, cervical revascularization technique in reference to the ICA lesion (anterograde vs retrograde), and antiplatelet therapy regimens (when stenting was performed) categorized as single, dual, and/or intravenous antiplatelet(s) administered immediately before, during, or after the endovascular therapy procedure. Treatment with intravenous thrombolysis was determined at the discretion of the treating clinician. All intracranial occlusions were treated using stent retriever and/or contact aspiration catheters. The endovascular and medical therapeutic interventions were performed according to each institution’s protocol with the patient under conscious sedation or general anesthesia and at the discretion of the interventionalists.

### Outcome Measures

The primary clinical outcome for the study was favorable functional outcome, measured as a 90-day mRS score of 0 to 2, obtained by board-certified vascular neurologists during a routinely scheduled clinical visit or by a certified nurse during a standardized telephone interview at each center. The primary safety outcome was sICH, as defined according to the European Collaborative Acute Stroke Study 3 criteria.^15^ Secondary outcomes included successful reperfusion (modified Thrombolysis in Cerebral Infarction [mTICI] score of 2b and 3, defined as antegrade reperfusion of previously more than half and completely occluded target artery territory, respectively),^16^ discharge mRS score, ordinal shift in 90-day mRS score, and 90-day mortality. Procedural complications were divided into hemodynamic impairment (bradycardia requiring atropine, hypertension requiring labetalol, and hypotension requiring vasopressors) and intracranial or extracranial complications at the time of treatment (vessel perforation, arterial dissection, access site complication requiring surgical repair or blood transfusion, intraprocedural mortality, and in-stent thrombosis).

### Statistical Analysis

Descriptive statistics were used to summarize continuous and categorical variables. We reported categorical variables as numbers (percentages) and continuous variables as means (SDs) or medians (IQRs). The Shapiro-Wilk test and histograms were used to assess normality of distributions. For the univariate analysis, we used 2-tailed, paired *t* tests or Wilcoxon rank-sum test for continuous variables and the χ^2^ or Fisher exact test for categorical variables, as needed.

Multivariable logistic regression was performed to evaluate the association between the extracranial ICA lesion treatment and the clinical and safety outcomes. The final models for both mRS scores at 90 days and sICH were selected by applying backward and forward stepwise selection procedures with a set of a priori covariates that included sex, intravenous thrombolysis, time from LKW, atrial fibrillation, NIHSS score, ASPECTS, antiplatelet use, high-volume center (a high-volume center was defined as treating ≥30 patients with TLs at a center within the study period), preprocedural ICA status, intracranial procedure, and reperfusion status. The selection procedure maintained the inclusion of stent and ASPECTS. An adjusted ordinal logistic regression model was also generated to estimate the odds of improved mRS scores at 90 days. When fitting all the multivariable models, we performed multiple imputations with chained equations (m = 10 imputations) under the assumption that missing data are missing at random. Finally, the adjusted odds ratios (aORs) and 95% CIs from multiple imputations were calculated using the Rubin rules of pooling.^17^ A sensitivity analysis using logistic regression and following similar steps was performed exclusively in patients with successful reperfusion mTICI scores of 2b and higher to better evaluate the association of CAS with functional outcome. Furthermore, we performed propensity score matching based on the baseline covariates that differed between the treatment groups, including sex, NIHSS score at presentation, antiplatelets regimens, and degree of cervical ICA stenosis. Propensity score matching was performed with a caliper of 0.2 times the SD of the propensity score logit, with an exposed-control ratio of 1:1. Quality of the matching process was assessed through balance analysis with standardized mean differences, where a value less than 0.1 was considered balanced.

Moreover, we compared the performance of our primary efficacy outcome logistic model to a gradient boosting model (a machine learning classifier demonstrated to have excellent performance in predicting categorical outcomes). We conducted an out-of-sample performance analysis on functional outcome at 90 days using the same covariates as the final logistic model. The tuning parameters of gradient boosting within each out-of-sample test, such as the minimum number of observations in the terminal nodes, the total number of trees to fit, and the shrinkage parameters, were selected through a 20-fold cross-validation. The purpose of the comparison was to collate the algorithm with our logistic regression model. We also evaluated the accuracy of probabilistic projections using the Brier score.

Last, we performed an exploratory analysis to evaluate the heterogeneity of CAS for prespecified subgroups of the primary efficacy outcome, including age, intravenous thrombolysis, etiology (atherosclerosis vs dissection), ASPECTS, antiplatelet use, technique (anterograde vs retrograde), preprocedural ICA lesion status (stenosis vs occlusion), and time from LKW (early vs late window). The models were adjusted by time from LKW, NIHSS, high-volume center, and reperfusion status. We similarly assessed heterogeneity of CAS for the primary safety outcome on prespecified variables, including ASPECTS, use of antiplatelets and heparin, and time from LKW (early vs late window). The models were adjusted for time from LKW and reperfusion status. The aORs (95% CIs) for the effect size of each group were computed. All the statistical analyses were considered significant at a 2-sided α≤.05. Statistical analyses were performed using R software, version 3.3.0 (R Foundation for Statistical Computing). Data analysis was performed from August 2021 to February 2022. Data will be made available on reasonable request from the corresponding author.

## Results

**Figure 1.**
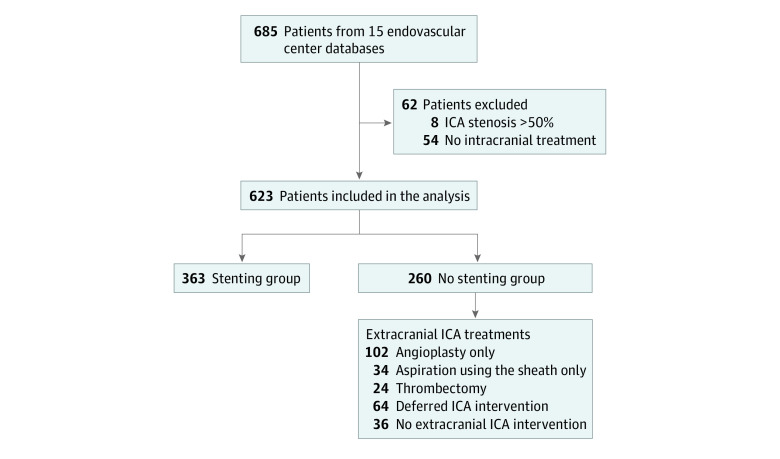
Flowchart of the Study Patients ICA indicates internal carotid artery.

Among the total of 685 patients, 62 patients were excluded (54 did not receive any intracranial treatment due to the absence of LVO in the angiography suite and 8 had preprocedural ICA stenosis <50%). Therefore, 623 patients (mean [SD] age, 67 [12.2] years; 406 [65.2%] male and 217 [34.8%] female) were included in the analysis, of whom 363 (58.4%) were in the CAS group and 260 (41.6%) were in the nonstenting group (Figure 1 and eFigure 1 in Supplement 1). Demographic and baseline characteristics of the 2 groups are presented in Table 1. The stenting group had a significantly higher number of men (253 [69.7%] vs 153 [58.8%]; *P* = .005). There were fewer patients with atrial fibrillation (38 [10.6%] vs 49 [19.2%]; *P* = .002), more patients with preprocedural cervical stenosis (90%-99%: 107 [32.2%] vs 42 [20.5%]; *P* < .001) and atherosclerotic disease (296 [82.0%] vs 194 [74.6%]; *P* = .003); and lower median (IQR) NIHSS scores (15 [10-19] vs 17 [13-21]; *P* < .001) in the stenting group compared with the nonstenting group. Smoking, median ASPECTS score, intravenous thrombolysis use, and stroke time metrics were similar among both groups.

**Table 1.** Baseline and Procedural Characteristics and Stroke Time Metrics Among Patients With Carotid Artery Stenting and Nonstenting^a^

Characteristic	Total (N = 623)	Carotid artery stenting group (n = 363)	Nonstenting group (n = 260)	*P* value
Age, median (IQR), y	68 (59-76)	67 (59-75)	68 (59-77)	.40
Sex				
Male	406/623 (65.2)	253/363 (69.7)	153/260 (58.8)	.005
Female	217/623 (34.8)	110/363 (30.3)	107/260 (41.2)	
Hypertension	447/616 (72.6)	267/361 (74)	180/255 (70.6)	.36
Hyperlipidemia	280/614 (45.6)	165/360 (45.8)	115/254 (45.3)	.89
Diabetes	164/615 (26.7)	97/361 (26.9)	67/254 (26.4)	.89
Atrial fibrillation	87/615 (14.1)	38/360 (10.6)	49/255 (19.2)	.002
Current smoker	155/613 (25.3)	92/358 (25.7)	63/255 (24.7)	.94
Previous stroke or TIA	96/615 (15.6)	58/359 (16.2)	38/257 (14.8)	.63
Coronary artery disease	118/616 (19.2)	72/360 (20)	46/256 (18.0)	.53
NIHSS score at admission, median (IQR) (n = 619)	16 (11-20)	15 (10-19)	17 (13-21)	<.001
NIHSS categorized				
Mild (score, ≤5)	47/619 (7.6)	38/360 (10.6)	9/259 (3.5)	<.001
Moderate (score, 6-15)	249/619 (40.2)	155/360 (43.1)	94/259 (36.3)	
Severe (score, ≥16)	322/619 (52.2)	167/360 (46.4)	156/259 (60.2)	
Intravenous thrombolysis treatment	266/622 (42.8)	146/363 (40.2)	120/259 (46.3)	.13
ASPECTS, median (IQR)	8 (7-9)	8 (7-9)	8 (7-10)	.50
Patients from high-volume centers	470 (80.1)	305 (84)	165 (73.7)	.002
Periprocedural antiplatelets regimen				
Single therapy	138/600 (23)	73/363 (20.1)	65/237 (27.4)	<.001
Dual therapy	211/600 (35.2)	145/363 (39.9)	66/237 (27.8)	
Intravenous therapy	174/600 (29)	139/363 (38.3)	35/237 (14.8)	
No therapy	77/600 (12.8)	6/363 (1.7)	71/237 (30)	
Cervical ICA stenosis, %				
50-69	20/537 (3.7)	5/332 (1.5)	15/205 (7.3)	<.001
70-89	76/537 (14.2)	47/332 (14.2)	29/205 (14.1)	
90-99	149/537 (27.7)	107/332 (32.2)	42/205 (20.5)	
100	292/537 (54.4)	173/332 (52.1)	119/205 (58)	
Postprocedural ICA stenosis, %				
50-69	72 (12.8)	16 (4.7)	56 (25.3)	<.001
70-89	23 (4.1%)	1 (0.3)	22 (10)	
90-99	19 (3.4)	6 (1.8)	13 (5.9)	
100	30 (5.3)	9 (2.6)	21 (9.5)	
Cervical ICA lesion treatment				
Anterograde	269/583 (46.1)	175/360 (48.6)	94/223 (42.2)	<.001
Concomitant	89/583 (15.3)	62/360 (17.2)	27/223 (12.1)	
Retrograde	152/583 (26.1)	123/360 (34.2)	29/223 (13.0)	
Not initially treated	72/583 (12.3)	0	72/223 (32.3)	
Cervical ICA lesion treatment with adjunctive aspiration	219/306 (38.7)	144/250 (41.1)	75/216 (34.7	.13
General anesthesia	208/623 (33.5)	122/363 (33.6)	86/258 (33.3)	.94
Etiology of cervical ICA lesion				
Atherosclerotic disease	490/621 (78.9)	296/361 (82.0)	194/260 (74.6)	.003
Dissection	66/621 (10.6)	41/361 (11.4)	25/260 (9.6)	
Oher	63/621 (10.1)	23/361 (6.4)	40/260 (15.4)	
Intracranial procedure other than MT				
Intracranial angioplasty alone	20/597 (3.4)	3/360 (0.8)	17/237 (7.2)	<.001
Intracranial stenting and angioplasty	82/597 (13.7)	75/360 (20.8)	7/237 (2.9)	
None	495/597 (82.9)	282/360 (78.3)	213 (89.9)	
Intra-arterial tPA treatment	30/605 (5)	12/353 (3.4)	18/252 (7.1)	.04
Time metrics, median (IQR), min				
Time from LKW to arterial puncture (n = 588)	351 (215-706)	350 (213-749)	352 (219-637)	.52
Door to arterial puncture (n = 605)	65 (28-109)	65 (31-109)	65 (26-109)	.89
Arterial puncture to reperfusion (n = 599)	56 (37-87)	58 (38-88)	54 (33-87)	.14

Abbreviations: ASPECTS, Alberta Stroke Program Early CT Score; ICA, internal carotid artery; LKW, last known well; MT, mechanical thrombectomy; NIHSS, National Institutes of Health Stroke Scale; TIA, transient ischemic stroke; tPA, tissue plasminogen activator.

^a^ Data are presented as number/total number (percentage) of patients unless otherwise indicated.

### Primary Clinical Outcome

**Figure 2.**
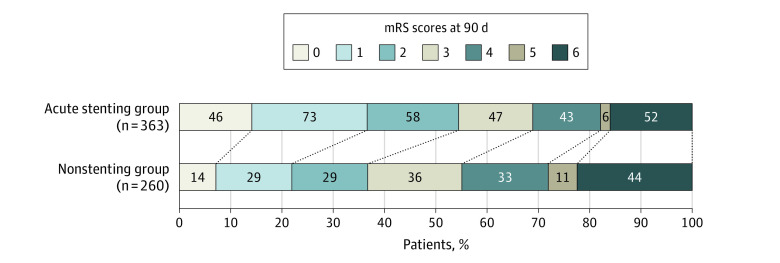
Distribution of 90-Day Modified Rankin Scale (mRS) Score Among Patients With Carotid Artery Stenting and Nonstenting Scores range from 0 to 6, with 0 indicating no symptoms; 1, no clinically significant disability; 2, slight disability (the patient is able to look after own affairs without assistance but unable to perform all previous activities); 3, moderate disability (patient requires some help but is able to walk unassisted); 4, moderately severe disability (patient is unable to attend to bodily needs without assistance and unable to walk unassisted); 5, severe disability (patient requires constant nursing care and attention); and 6, death.

There was a significantly increased number of patients with favorable functional outcome at 90 days in the CAS group compared with the nonstenting group (177 [54.5%] vs 72 [36.7%]; *P* < .001). After adjusting for time from LKW, NIHSS scores, ASPECTS, high-volume center, and reperfusion status, patients treated with CAS had 1.7 times higher odds of favorable outcome compared with the nonstenting group (aOR, 1.67; 95% CI, 1.20-2.40; *P* = .007) (Table 2; eTable 1 in Supplement 1). Similarly, the CAS group had a 46% increase in the odds of having a favorable shift in mRS scores at 90 days when compared with the nonstenting group, after adjusting for the same confounders (aOR, 1.46; 95% CI, 1.02-2.10; *P* = .04) (Figure 2). Moreover, the significant association of CAS with favorable functional outcome at 90 days persisted in the propensity score matching (aOR, 2.10; 95% CI, 1.43-3.12; *P* < .001) and when the analysis was restricted to include only patients with mTICI scores of 2b and higher (aOR, 1.68; 95% CI, 1.06-2.66; *P* = .03) (eTable 2 and eFigure 2 in Supplement 1). The out-of-sample performance analysis in our study found that the primary logistic outcome model had a mean (SD) area under the curve of 0.73 (0.0695) (Brier score, 0.21), whereas the gradient boosting model had a mean (SD) area under the curve of 0.697 (0.0647) (Brier score, 0.22), indicating similar performance (eTable 3 in Supplement 1).

**Table 2.** Clinical and Radiographic Outcomes Among Patients With Carotid Artery Stenting and Nonstenting Groups

Outcome	Sample size	No. (%) of patients			Unadjusted		Adjusted	
		Total	Carotid artery stenting group	Nonstenting group	OR (95% CI)	*P* value	OR (95% CI)	*P* value
Primary outcomes								
90-d mRS score 0-2^a^	554	249 (47.8)	177 (54.5)	72 (36.7)	1.96 (1.39-2.77)	<.001	1.67 (1.2-2.4)	.007
Symptomatic ICH^b^	538	28 (5.2)	18 (5.5)	10 (4.8)	1.02 (0.5-2.1)	.96	0.9 (0.46-1.94)	.87
Secondary outcomes								
mTICI score ≥2b^c^	548	477 (86.7)	306 (90.5)	171 (80.7)	2.3 (1.4-3.7)	.001	1.7 (1.02-3.6)	.002
Discharge mRS score 0-2^d^	443	114 (25.7)	74 (28.9)	40 (21.4)	1.6 (1.08-2.28)	.02	1.2 (0.8-1.8)	.41
Mortality at 90 d^e^	521	99 (18.4)	52 (16.0)	44 (22.4)	0.64 (0.42-0.97)	.03	0.78 (0.5-1.2)	.27
Periprocedural hemodynamic impairment^f^	NA	56 (15.4)	43 (11.8)	13 (5.0)	NA	.01	NA	NA
Intracranial and extracranial complications at time of treatment^g^	NA	34 (6.2)	18 (6.1)	13 (6.4)	NA	.92	NA	NA

Abbreviations: ICH, intracranial hemorrhage; mRS, modified Rankin Scale; mTICI, modified Thrombolysis in Cerebral Infarction; NA, not applicable; OR, odds ratio.

^a^ Model adjusted for last known well, National Institutes of Health Stroke Scale score, Alberta Stroke Program Early CT Score, high-volume center, and mTICI score.

^b^ Model adjusted for last known well, Alberta Stroke Program Early CT Score, and mTICI score.

^c^ Model adjusted for extracranial preprocedure stenosis and other procedures (intracranial plasty, intracranial stenting, or both vs other procedures).

^d^ Model adjusted for last known well, National Institutes of Health Stroke Scale score, Alberta Stroke Program Early CT Score, and mTICI score.

^e^ Model adjusted for National Institutes of Health Stroke Scale score and mTICI score.

^f^ Sample sizes for periprocedural hemodynamic impairment are as follows: bradycardia requiring atropine: total, 21; stenting group, 17; and nonstenting group, 4; hypertension requiring labetalol: total, 2; stenting group, 1; and nonstenting group, 1; and hypotension requiring vasopressors: total, 33; stenting group, 25; and nonstenting group, 8.

^g^ Sample sizes for intracranial and extracranial complications at time of treatment are as follows: vessel perforation, 4; arterial dissection, 5; intraprocedural mortality, 10; device failure, 13; and embolization into different territory, 2.

### Primary Safety Outcome

The rate of sICH was comparable between the 2 groups (18 [5.5%] vs 10 [4.8%], *P* = .96). No significant difference was found between the 2 groups (aOR, 0.90; CI, 0.46-1.94; *P* = .87) after adjusting for time from LKW, ASPECTS, and reperfusion status (Table 2; eTables 1 and 5 in Supplement 1). Similarly, no significant association was observed after propensity score matching (aOR, 1.40; 95% CI, 0.40-4.60; *P* = .62) or when the analysis was restricted to include only patients with mTICI scores of 2b and higher (aOR, 0.74; 95% CI, 0.27-2.03; *P* = .59) (eTable 2 and eFigure 2 in Supplement 1).

### Secondary Outcomes

More patients had successful reperfusion (mTICI score ≥2b) in the CAS group compared with the nonstenting group (306 [90.5%] vs 171 [80.7%]; aOR, 1.70 [95% CI, 1.02-3.60]; *P* = .002), after adjusting for the type of procedure (CAS and nonstenting) and preprocedural extracranial ICA stenosis status. More patients underwent periprocedural hemodynamic impairment in the CAS group than the nonstenting group (43 [11.8%] vs 13 [5.0%]; *P* = .01), whereas the remaining procedural complications were similar between the 2 groups (Table 2 and eTable 4 in Supplement 1).

No significant association was found for the discharge mRS score of 0 to 2 (aOR, 1.20; 95% CI, 0.80-1.80; *P* = .41) and 90-day mortality (aOR, 0.78; 95% CI, 0.50-1.20; *P* = .27) among the 2 groups (Table 2; eTable 1 in Supplement 1). Although on the propensity score–matched analysis stenting was observed to be associated with an increased odds of discharge mRS scores of 0 to 2 (aOR, 1.70; 95% CI, 1.09-2.52; *P* = .02), no significant association was observed for favorable functional outcome and mortality at 90 days (eFigure 2 in Supplement 1). Similarly, no association was observed for discharge mRS scores of 0 to 2 or 90-day mortality when the analysis was restricted to include only patients with mTICI scores of 2b and higher (eTable 2 in Supplement 1).

### Heterogeneity of CAS on Prespecified Subgroups

**Figure 3.**
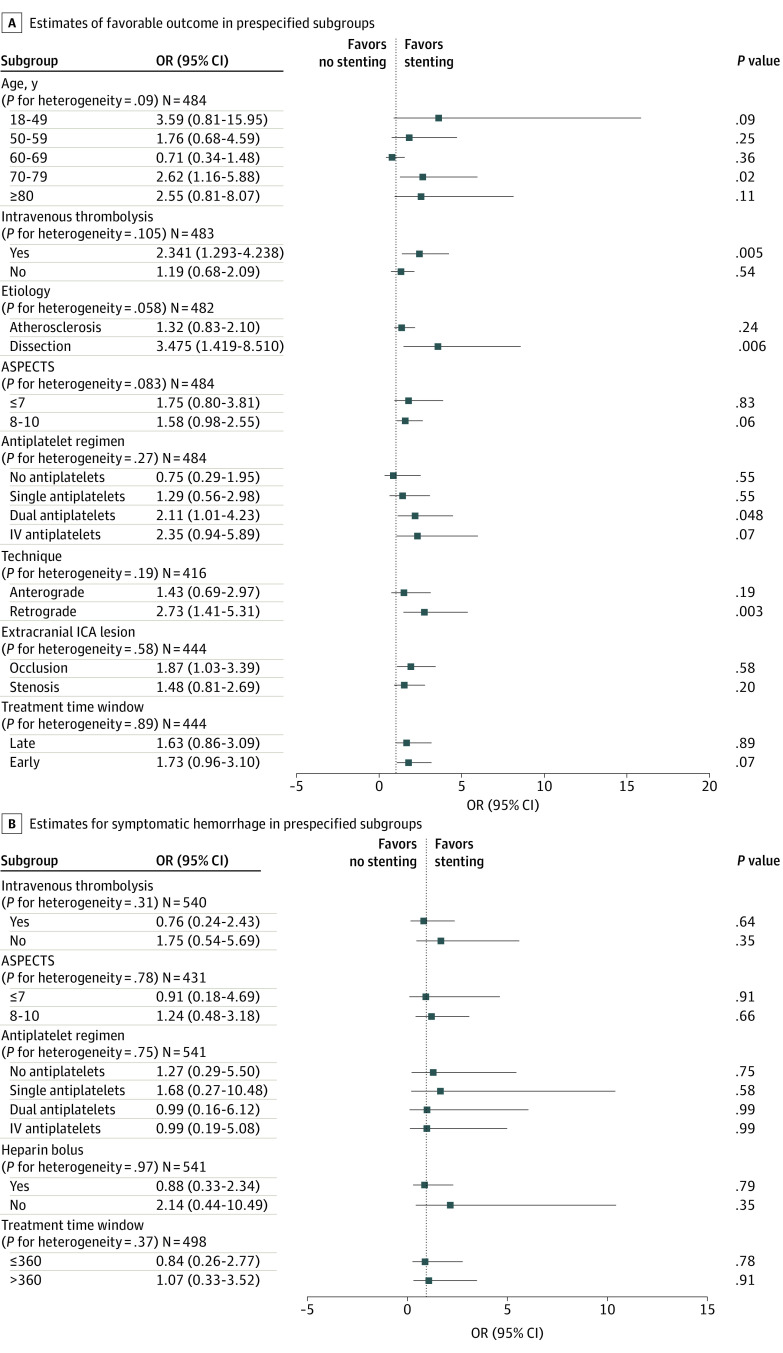
Adjusted Treatment Estimates of Favorable Outcomes and Symptomatic Intracranial Hemorrhage ASPECTS indicates Alberta Stroke Program Early CT Score; IV, intravenous; OR, odds ratio.

Our study did not observe any heterogeneity for the primary outcome of 90-day functional status across the prespecified variables, including age, intravenous thrombolysis, etiology (dissection vs atherosclerosis) or type (stenosis vs occlusion) of cervical lesion, ASPECTS, use of antiplatelet regimens (single, dual, or intravenous), procedural techniques (anterograde vs retrograde), or time from LKW (early vs late). For the subgroup analyses, we observed that CAS favors functional independence for the age group 70 to 79 years (aOR, 2.62; CI, 1.16-5.88; *P* = .02), use of intravenous thrombolysis (aOR, 2.30; 95% CI, 1.29-4.24; *P* = .005), etiology (dissection: aOR, 3.50; 95% CI, 1.42-8.50; *P* = .006), and technique (anterograde: aOR, 2.70; 95% CI, 1.41-5.30; *P* = .003) (Figure 3A). Moreover, there was no evidence of heterogeneity observed for sICH across the prespecified variables, including ASPECTS; use of thrombolysis, heparin, or antiplatelets; or time from LKW (Figure 3B).

## Discussion

This multicenter, international cross-sectional study of individual patients identified the optimal management of TLs among patients with acute ischemic stroke. Our observations indicate that CAS of the extracranial ICA lesion was associated with significantly increased odds of successful reperfusion and better functional outcomes at 3 months without increasing the hemorrhage and mortality rates.

Our findings are consistent with several previously published cohorts that reported better clinical outcomes after CAS.^6,7^ Some reports^18,19^ found that stenting is associated with an increased recanalization rate, facilitated by restoration of the carotid artery blood flow and providing an ease of access to the intracranial lesion. However, because of the increased risk of in-stent thrombosis, use of combination antiplatelets (in addition to heightened risk of reperfusion injury) may increase the risk of intracranial hemorrhage.^18,19^ Our multicenter study observed that stenting was associated with increased odds of successful revascularization and favorable functional outcomes at 3 months, whereas clinically relevant hemorrhagic complications were similar across both groups. These results complement previously published reports^7,20^ and indicate that CAS is a safe treatment and may be associated with better outcomes when compared with angioplasty or medical management in patients with acute ischemic stroke with TLs.

We also observed that patients in the CAS group had significantly lower median NIHSS scores and a smaller proportion of patients with atrial fibrillation at admission compared with the nonstenting group. Although these differences in the baseline characteristics at presentation were small, they may have influenced the proceduralist’s decision to opt for stenting. Among patients with milder strokes (and presumably smaller infarct volumes), we would anticipate greater comfort among proceduralists with dual antiplatelet administration because the risk of hemorrhage would be attenuated. On the other hand, the history of atrial fibrillation may have confounded the clinician’s assessment of stroke mechanism or management strategy and could have motivated the clinician to prefer anticoagulation rather than stenting with combination antithrombotic therapy. Despite those baseline differences, CAS effect size associated with functional outcome was unchanged after adjusting for those confounders. Moreover, although increasing age is associated with an increased risk of complications, we did not observe differences in the rates of hemorrhage, although a stronger association of CAS was observed for those aged 70 to 79 years. This finding may reflect the proceduralist’s careful selection of patients and the use of newer improved embolic protection devices in this population.^21^ It is also possible, as observed in the Carotid Revascularization Endarterectomy vs Stenting Trial,^21^ a randomized clinical trial that compared CAS and carotid endarterectomy in asymptomatic and symptomatic isolated carotid stenosis, that populations older than 70 years may have a differential beneficial effect from stenting in TLs. However, further investigation is warranted to elucidate the association of age with CAS treatment outcomes.

Among patients who underwent CAS in this cohort, we also observed that 49% of the patients received stents before proceeding to intracranial LVO thrombectomy (anterograde approach). This finding suggests that most proceduralists might have needed to proximally intervene to gain access to the distal lesion or may have preferred to stabilize the cervical lesion first. An anterograde approach has been argued to facilitate the flow of blood and may precipitate distal perfusion via collateral circulation, thereby reducing the rates of stroke recurrence.^22,23^ Moreover, it provides better technical support for accessing the intracranial occlusion, especially when there is high-grade stenosis or complete occlusion of the cervical segment. On the other hand, initial treatment of the intracranial occlusion has been reported to decrease the reperfusion times while preventing the risk of distal embolization.^9^ Although both techniques are widely used, factors such as stroke severity at presentation, extent of cervical lesion stenosis, and collateral vessels may influence the interventionalist’s decision. Although, in our study, we did not observe any heterogeneity for the treatment approaches and both the anterograde and retrograde techniques favored better functional outcome at 3 months, the odds were nearly 3-fold higher with the retrograde approach, which is in line with a recently published meta-analysis showing that the retrograde approach is significantly associated with mRS scores of 2 or lower at 90 days.^24^

The underlying etiology of the cervical lesion is also an important consideration. Although most lesions are atherosclerotic in nature, dissections and embolic occlusions have also been reported.^3,25^ In our cohort, most of the lesions were atherosclerotic (78.9%), with fewer dissections (10.6%) and embolic occlusions (10.1%). Irrespective of the lesion etiology, stenting has been associated with similar clinical and functional outcomes and with decreased risk of recurrent strokes in the setting of an underlying atherosclerotic disease.^25,26^ Moreover, in our exploratory analysis, we observed that CAS favors functional outcome in patients with cervical ICA dissection. Although these findings may be due to etiological characteristics, patients with cervical ICA dissections tend to be younger and have better neurological and functional outcomes than patients with atherosclerotic disease.^27,28^

The most feared complication is the risk of hemorrhage when using a stent that requires antiplatelet therapy. In our study, the hemorrhagic rate of CAS was similar to that of the nonstenting group. In addition, we also observed a stronger association of stenting with the functional outcome within the subgroup of patients using dual antiplatelet medications. These findings reflect previously published results from the TITAN (Thrombectomy in Tandem Occlusions) registry, which demonstrated that stenting along with the use of antithrombotic agents was associated with increased rates of recanalization and better functional outcomes at 90 days.^6^ Moreover, in our subgroup analysis, we observed 2-fold increased odds of CAS on functional outcomes in patients with prior intravenous thrombolytic therapy, whereas no significant difference was observed in the rates of symptomatic hemorrhage, among these patients. These results are similar to those of a recently published meta-analysis^24^ observing similar rates of symptomatic hemorrhage among patients with TLs receiving CAS with or without intravenous thrombolytic therapy. Although factors such as delayed time for endovascular therapy initiation or atherosclerotic nature of TLs with varied degrees of clot burden may reflect this finding, larger studies are warranted to understand the role of thrombolytic therapy in TLs.

### Limitations

These observations should be interpreted with reference to the following limitations. There is an inherent potential for selection bias due to retrospective study design. Patient selection was based on practitioner preference and was not standardized or randomized, thus affecting overall outcomes and the decision for treatment approaches. In addition, the clinical and imaging data provided by the discrete centers was not centrally monitored or adjudicated by a core imaging laboratory, which may result in reporting bias in rates of successful revascularization and the extent of extracranial occlusion. Other important considerations, including anatomical variations, vessel tortuosity, and concerns on crossing a carotid stent with an intermediate catheter if an anterograde approach is used, may have resulted in altering the proceduralist’s techniques. Finally, the lack of an adequate sample size calculation may result in failure to detect some differences in the comparison groups, and the presence of missing data on some variables may influence the estimate calculations, despite performing multiple imputations.

Although this study adds to the literature, several factors, such as type, duration, and combination of various medications (aspirin, clopidogrel, tirofiban, prasugrel, ticagrelor, cangrelor, glycoprotein IIb/IIIa inhibitors, and others), time and mode of medication administration (oral or intravenous and before, during, or after the procedure), use of intravenous heparin (along with antiplatelet regimens), type of stents (single or dual layer), and/or use of embolic protection devices, may help in standardizing the complex management of TLs for the prospective randomized comparison of endovascular approaches.

## Conclusions

The findings of this multicenter, international cross-sectional study suggest that stenting of the extracranial cervical lesion during MT for intracranial LVO may be an effective and safe treatment in patients with acute ischemic stroke with TLs. Carotid artery stenting was associated with significantly increased odds of favorable outcome and successful revascularization without increasing the hemorrhage and mortality rates.

## Notes

Supplement 1. **eTable 1.** Clinical and Radiographic Outcomes Among Patients With Acute Carotid Stenting and Non-stenting Groups Before and After Multiple Imputations **eTable 2.** Clinical and Radiographic Outcomes Among Patients With Acute Carotid Stenting and Non-stenting in Patients With Thrombolysis in Cerebral Infarction (TICI)>2B **eTable 3.** Comparison of Performances of Predictive Models **eTable 4.** Reported Complications and Functional Outcomes at 90 Days in the Delayed Stenting Group **eTable 5.** Symptomatic Intracranial Hemorrhage (sICH) Using Different Definitions **eFigure 1.** Acute Stenting of the Extracranial Carotid Artery **eFigure 2.** Clinical and Radiographic Outcomes Among Patients With Acute Carotid Stenting and Non-stenting in Patients After Matching Using Propensity Score (PS) Matching

Supplement 2.
Data Sharing Statement
