# Supplementary material for: Functional and Safety Outcomes of Carotid Artery Stenting and Mechanical Thrombectomy for Large Vessel Occlusion Ischemic Stroke With Tandem Lesions
Source: JAMA Netw Open. 2023 Mar 1;6(3):e230736. doi: 10.1001/jamanetworkopen.2023.0736 (PMC9978940; doi:10.1001/jamanetworkopen.2023.0736)
Supplement: Supplement 1. — eTable 1. Clinical and Radiographic Outcomes Among Patients With Acute Carotid Stenting and Non-stenting Groups Before and After Multiple Imputations eTable 2. Clinical and Radiographic Outcomes Among Patients With Acute Carotid Stenting and Non-stenting in Patients With Thrombolysis in Cerebral Infarction (TICI)>2B eTable 3. Comparison of Performances of Predictive Models eTable 4. Reported Complications and Functional Outcomes at 90 Days in the Delayed Stenting Group eTable 5. Symptomatic Intracranial Hemorrhage (sICH) Using Different Definitions eFigure 1. Acute Stenting of the Extracranial Carotid Artery eFigure 2. Clinical and Radiographic Outcomes Among Patients With Acute Carotid Stenting and Non-stenting in Patients After Matching Using Propensity Score (PS) Matching [file jamanetwopen-e230736-s001.pdf]

Supplementary Online Content

Farooqui M, Zaidat OO, Hassan AE, et al. Functional and safety outcomes of carotid artery stenting and mechanical thrombectomy for large vessel occlusion ischemic stroke with tandem lesions. *JAMA Netw Open*. 2023;6(2):e230736. doi:10.1001/jamanetworkopen.2023.0736

eTable 1. Clinical and Radiographic Outcomes Among Patients With Acute Carotid Stenting and Non-stenting Groups Before and After Multiple Imputations

eTable 2. Clinical and Radiographic Outcomes Among Patients With Acute Carotid Stenting and Non-stenting in Patients With Thrombolysis in Cerebral Infarction (TICI) >2B

eTable 3. Comparison of Performances of Predictive Models

eTable 4. Reported Complications and Functional Outcomes at 90 Days in the Delayed Stenting Group

eTable 5. Symptomatic Intracranial Hemorrhage (sICH) Using Different Definitions

eFigure 1. Acute Stenting of the Extracranial Carotid Artery

eFigure 2. Clinical and Radiographic Outcomes Among Patients With Acute Carotid Stenting and Non-stenting in Patients After Matching Using Propensity Score (PS) Matching

This supplementary material has been provided by the authors to give readers additional information about their work.

© 2023 Farooqui M et al. *JAMA Network Open.*

eTable 1: Clinical and radiographic outcomes among patients with acute carotid stenting and non-stenting groups before and after multiple imputations.

	Before imputation		After imputation	
**Primary Outcomes**	aOR (95% CI)	P Value	aOR (95% CI)	P Value
*90-d mRS score 0-2 No. %, (N=521)	1.74 (1.1-2.7)	0.015	1.67 (1.2-2.4)	0.007
** Symptomatic ICH No. %, (N=538)	0.8 (0.37-1.83)	0.63	0.9 (0.46-1.94)	0.87
**Secondary Outcomes**				
+ mTICI score ≥2b, No. %, (N=550)	2.2 (1.34-3.67)	<0.001	1.7 (1.02-3.6)	0.002
++Discharge mRS score 0-2 No.%, (N=443)	1.2 (0.74-1.91)	0.47	1.2 (0.8-1.8)	0.41
+++ Mortality at 90 days, No. %, (N=521)	0.79 (0.49-1.27)	0.32	0.78 (0.5-1.2)	0.27

*Model adjusted for last known well, NIHSS, ASPECTS, high volume center and TICI.

**Model adjusted for last known well, ASPECTS, and TICI.

+Model adjusted for extracranial pre-procedure stenosis and other procedures (intracranial plasty, intracranial stenting, or both vs other procedures)

++Model adjusted for last known well, NIHSS, ASPECTS, and TICI.

+++ Model adjusted for NIHSS and TICI.

© 2023 Farooqui M et al. *JAMA Network Open.*

eTable 2: Clinical and radiographic outcomes among patients with acute carotid stenting and non-stenting in patients with thrombolysis in cerebral infarction (TICI) >2B.

	Total (N=548) N (%)	Stenting group (N=332) N (%)	Non Stenting Group (N=216) N (%)	Adjusted	
**Primary Outcomes**				aOR (95% CI)	P Value
*90-d mRS score 0-2	255 (52.6)	173 (58.4)	82 (43.4)	1.68 (1.06-2.66)	0.026
** Symptomatic ICH	24 (4.4)	14 (4.2)	10 (4.7)	0.74 (0.27-2.03)	0.59
**Secondary Outcomes**					
++Discharge mRS score 0-2	159 (29.6)	108 (32.8)	51 (24.5)	1.31 (0.82-2.1)	0.26
+++ Mortality at 90 days	99 (18.4)	52 (16.0)	44 (22.4)	0.84 (0.51-1.41)	0.51

*Model adjusted for last known well, NIHSS, ASPECTS, high volume center and TICI.

**Model adjusted for last known well, ASPECTS, and TICI.

++Model adjusted for last known well, NIHSS, ASPECTS, and TICI.

+++ Model adjusted for NIHSS and TICI.

© 2023 Farooqui M et al. *JAMA Network Open.*

eTable 3: Comparison of performances of predictive models

Model	Mean AUC	SD	Brier Score
Logistic Regression	0.7308	0.0695	0.21
Gradient Boosting	0.698	0.0647	0.22

© 2023 Farooqui M et al. *JAMA Network Open.*

eTable 4: Reported complications and functional outcomes at 90-days in the delayed stenting group.

Delayed Stenting group			
Time	< 24 hours	24 - 48 hours	> 48 hours
Total cases	N=16	N=6	N=52
Complications	Vasospasm: 1<br>Petechial hemorrhage: 1<br>PH1: 1<br>IVH: 1	Petechial hemorrhage: 1<br>SAH: 1<br>Re-occlusion: 2	Re-occlusion: 1<br>PH1: 6<br>PH 2: 1<br>Petechial hemorrhage: 9<br>SAH: 1
mRS 0-2	9 (56.3%)	4 (66.7%)	26 (50%)
*Periprocedural hemodynamic impairment	1 (6.3%)	1 (16.6%)	5 (9.6%)
#Intra/Extra cranial complications at time of treatment	2 (12.5%)	1 (16.6%)	4 (7.7%)

mRS= modified Rankin Scale

*Periprocedural hemodynamic impairment: Bradycardia requiring atropine (2), Hypertension requiring labetalol (1), Hypotension requiring vasopressor (4)

#Intra/Extra cranial Complications at Time of Treatment: Vessel perforation (1), Arterial dissection (2), Intraprocedural mortality (1), Device failure (2), Embolization into different territory (1)

© 2023 Farooqui M et al. *JAMA Network Open.*

eTable 5. Symptomatic intracranial hemorrhage (sICH) using different definitions.

sICH	Stenting Group N (%)	Non-Stenting Group N (%)	Total
PH2 and NIHSS increase ≥4[a]	2 (1.2%)	1 (0.6%)	3
Any ICH and NIHSS increase ≥1[b]	22 (3.2%)	19 (2.4%)	41

[a] Safe Implementation of Thrombolysis in Stroke-Monitoring Study (SITS-MOST)

[b] National Institute of Neurological Disorders and Stroke (NINDS)

© 2023 Farooqui M et al. *JAMA Network Open.*

eFigure 1: Acute stenting of the extracranial carotid artery.

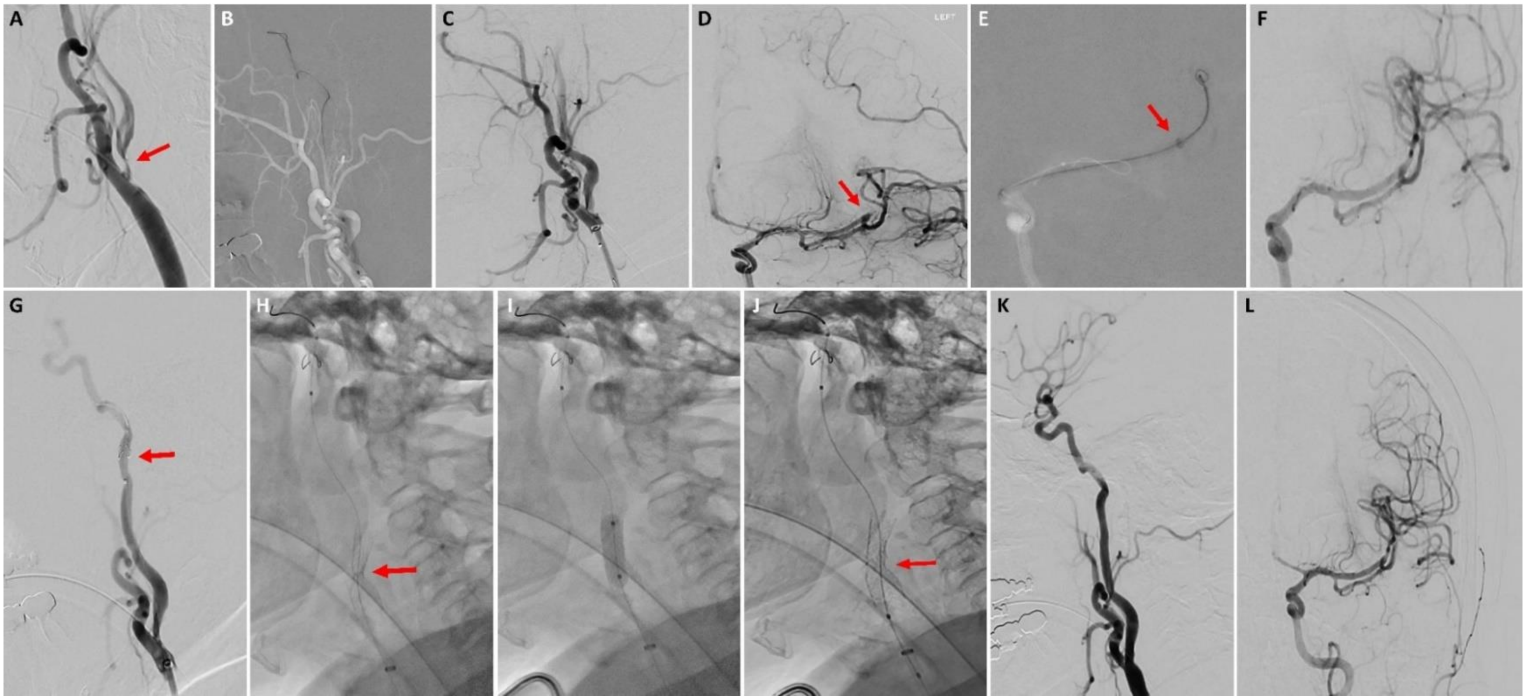

Figure (**A**-**L**). Case depicting a patient with a tandem lesion treated with mechanical thrombectomy followed by acute stenting of the extracranial carotid artery. (**A**) Initial digital subtraction angiography (DSA) lateral view image of the left common carotid showing severe flow-limiting stenosis (red arrow) at the origin of the left internal carotid artery (ICA). (**B**) Road map image showing the performance of balloon angioplasty of the stenotic segment of the ICA. (**C**) Control DSA run post-balloon angioplasty showing partial improvement of the original stenosis. (**D**) Anterior-posterior DSA view showing an intracranial occlusion at the proximal M2 segment of the middle cerebral artery (red arrow). (**E**) Road map image depicting the distal location of the microcatheter and intermediate catheter at the origin of the clot to perform mechanical thrombectomy through aspiration technique. (**F**) Control DSA after thrombectomy showing complete reperfusion (TICI 3) of the M2 segment. (**G**) DSA showing the deployment of the embolic protection device (EPD) at the distal cervical segment of the ICA (red arrow). Stent placement at the stenotic segment was performed. (**H**) Native fluoroscopic lateral view image showing the position of a fully deployed stent. Mild stenosis of the stent was noted (red arrow). (**I**) Post-stent balloon angioplasty to improve the lumen of the stent. (**J**) Control native fluoroscopic lateral view image after angioplasty showing improvement in the caliber of the lumen of the stent. (**K**) Final control DSA run showing an improvement in the caliber of the vessel at the origin of the ICA. Adequate antegrade flow was noted. (**L**) Final control run showing complete reperfusion of the intracranial vasculature.

© 2023 Farooqui M et al. *JAMA Network Open.*

eFigure 2: Clinical and radiographic outcomes among patients with acute carotid stenting and non-stenting in patients after matching using propensity score (PS) matching.

	Unadjusted			PS−adjusted		
Outcome	OR	95% CI	p−value	OR	95% CI	p−value
90−days mRS	2.0	1.40 − 2.72	<0.001	2.1	1.43 − 3.12	<0.001
Discharge mRS	1.7	1.16 − 2.45	0.007	1.7	1.09 − 2.52	0.019
mTICI 2b−3	2.2	1.34 − 3.59	0.002	2.2	0.98 − 4.92	0.057
90−days Mortality	0.7	0.44 − 1.02	0.059	0.7	0.44 − 1.02	0.059
sICH	1.0	0.00 − 1.00	1.68	1.4	0.40 − 4.60	0.621

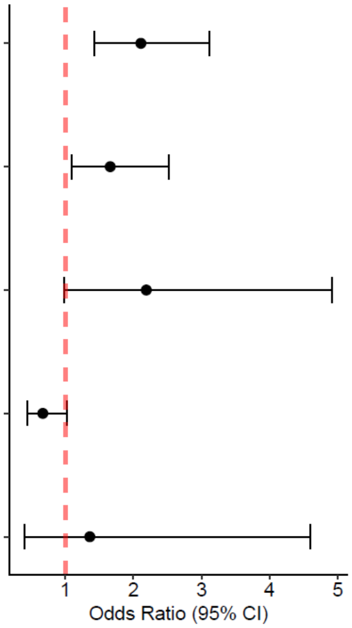

*Propensity score (PS) matching based on sex, race, NIHSS at admission, Periprocedural antiplatelets, and cervical ICA stenosis.

© 2023 Farooqui M et al. *JAMA Network Open.*
