# Supplementary material for: Functional and Safety Outcomes of Carotid Artery Stenting and Mechanical Thrombectomy for Large Vessel Occlusion Ischemic Stroke With Tandem Lesions
Source: JAMA Netw Open. 2023 Mar 1;6(3):e230736. doi: 10.1001/jamanetworkopen.2023.0736 (PMC9978940; doi:10.1001/jamanetworkopen.2023.0736)
Supplement: Supplement 2. — Data Sharing Statement [file jamanetwopen-e230736-s002.pdf]

Data Sharing Statement

Farooqui. Functional and Safety Outcomes of Carotid Artery Stenting and Mechanical Thrombectomy for Large Vessel Occlusion Ischemic Stroke With Tandem Lesions. *JAMA Netw Open*. Published March 01, 2023. doi:10.1001/jamanetworkopen.2023.0736

Data
Data available: Yes
Data types: Deidentified participant data
How to access data: Data will be made available from the corresponding author upon reasonable request
When available: With publication

Supporting Documents
Document types: None

Additional Information
Who can access the data: Data will be made available for the researchers upon reasonable request
Types of analyses: For a specified purpose
Mechanisms of data availability: Data will be made available after signed agreement
